# Exploring the Interactions of Soybean 7S Globulin with Gallic Acid, Chlorogenic Acid and (−)-Epigallocatechin Gallate

**DOI:** 10.3390/foods12214013

**Published:** 2023-11-02

**Authors:** Siduo Zhou, Ling Meng, Yanfei Lin, Xueqian Dong, Mingsheng Dong

**Affiliations:** 1 Shandong Food Ferment Industry Research & Design Institute, Qilu University of Technology (Shandong Academy of Sciences), Jinan 250013, China; zhousiduo@qlu.edu.cn; 2 College of Food Science and Technology, Nanjing Agricultural University, Nanjing 210095, China; 3 School of Public Health, Shandong First Medical University, Shandong Academy of Medical Sciences, 6699 Qingdao Road, Jinan 250117, China

**Keywords:** soybean 7S globulin, polyphenols, interaction, fluorescence spectroscopy, isothermal titration calorimetry, molecular docking

## Abstract

In this study, the noncovalent interaction mechanisms between soybean 7S globulin and three polyphenols (gallic acid (GA), chlorogenic acid (CA) and (−)-epigallocatechin gallate (EGCG)) were explored and compared using various techniques. Fluorescence experiments showed that GA and EGCG had strong static quenching effects on 7S fluorescence, and that of CA was the result of multiple mechanisms. The interactions caused changes to the secondary and tertiary structure of 7S, and the surface hydrophobicity was decreased. Thermodynamic experiments showed that the combinations of polyphenols with 7S were exothermic processes. Hydrogen bonds and van der Waals forces were the primary driving forces promoting the binding of EGCG and CA to 7S. The combination of GA was mainly affected by electrostatic interaction. The results showed that the structure and molecular weight of polyphenols play an important role in their interactions. This work is helpful for developing products containing polyphenols and soybean protein.

## 1. Introduction

Soybean is an important food crop species, and soybean products are among the richest sources of protein in plant-based diets. In Asia, many soybean-based foods have been consumed for many years, such as tofu, fermented bean curd, and tempeh, which still feature in people’s daily diet as the main raw material of homestyle dishes or side dishes. Soybean protein is a kind of high-quality protein from plants. Soybean protein is easy to digest and contains essential amino acids needed by the human body. In addition, soybean protein is often used as a food additive because of its good processability and low economic cost. Soybean 7S globulin, also known as β-conglycinin, is an important storage protein in soybean, accounting for approximately 30–46% of the total soybean protein content [1]. It has a molecular weight of approximately 150–180 kDa and is a trimeric protein composed of three subunits, namely, α, α’ and β [2]. On the basis of its complex tertiary and quaternary structures, 7S has an important impact on the function and quality of soybean protein, such as its emulsifying and foaming properties. In addition, by feeding mice and rats, previous researchers found that 7S also has a variety of potentially beneficial physiological effects, such as lowering blood fat content, blood glucose level and blood pressure [3,4,5].

**Figure 1:**
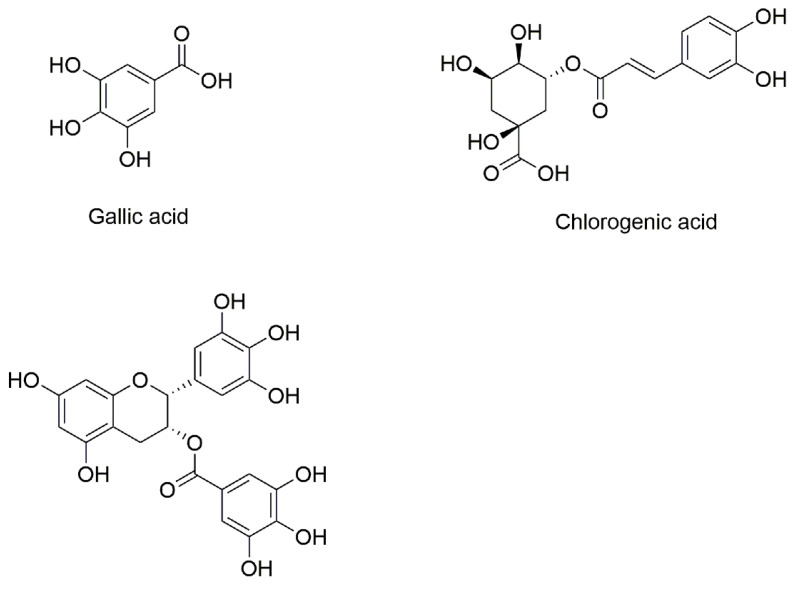
Chemical structure of gallic acid, chlorogenic acid and (−)-epigallocatechin gallate.

Polyphenols have received increasing attention for their diverse biological activities such as anti-tumor, anti-bacterial and anti-atherosclerotic properties, as well as for their beneficial effects on human health such as delaying or preventing oxidative damage from reactive oxygen species [6]. Polyphenols are primarily derived from plant-based foods and beverages, such as fruits and teas. Polyphenols contain at least one aromatic ring with one or more hydroxyl groups. Different types of carbon skeletons and different numbers of hydroxyl groups can result in polyphenols having different physicochemical properties and physiological activities. GA, CA and EGCG are three common polyphenols found in the daily diet. Figure 1 shows the chemical structures of GA, CA and EGCG.

Proteins and polyphenols are important food components, and non-covalent interactions between proteins and polyphenols are widely present in food systems. With the increasing demand for functional foods, the impact of the interaction between polyphenols and proteins on the functional properties of proteins has attracted attention in recent years.

Non-covalent modifications have been found to be a safe and effective way to improve the functional properties of proteins and the bioavailability of polyphenols [7]. Studying the interaction mechanisms between proteins and polyphenols at the molecular level is useful and very important for exploring the functional and physicochemical properties of proteins as well as the nutritional changes of foods during the dietary process [8].

In this study, soybean 7S globulin and three polyphenols (GA, CA and EGCG) with different structures were used as research objects to explore the mechanism of interaction between polyphenols and soybean protein. Multispectral methods, such as fluorescence spectroscopy and circular dichroism (CD), as well as molecular docking techniques were used to study and compare the mechanisms and differences in the interactions between the three polyphenols and 7S. Additionally, isothermal titration calorimetry (ITC) was also performed to characterize the thermodynamic parameters during the interactions between polyphenols and 7S. The results of this study will help us to further understand the interaction mechanism between soy protein and polyphenols in the human diet or food system. The results can also provide some reference value for the design and development of functional foods containing soybean protein and polyphenols.

## 2. Materials and Methods

### 2.1. Materials

Soybeans were purchased from supermarkets. GA (purity ≥ 98%, PubChem CID: 370), CA (purity ≥ 98%, PubChem CID: 1794427) and EGCG (purity ≥ 98%, PubChem CID: 65064) were purchased from Yuanye Biotechnology Co., Ltd. (Shanghai, China). 1-Anilino-8-naphthalenesulfonic acid (ANS) was purchased from TCI Development Co., Ltd. (Shanghai, China). All other analytical reagent-grade chemicals were purchased from Sinopharm Chemical Reagent Co., Ltd. (Shanghai, China).

### 2.2. Preparation of 7S Globulin

The soybeans were crushed and passed through a 40-mesh sieve. The soybean flour was defatted using *n*-hexane (material/liquid = 1:3 (*w*/*v*)) for 2 h at room temperature, and this process was repeated twice. After centrifugation (8000× *g*, 15 min, 4 °C), the defatted soybean powder was collected and dried at a low temperature until use.

The 7S globulin was extracted according to a modified version of a method reported by Ren et al. [9]. The defatted soy flour (10 g) was dissolved in 15 volumes of distilled water whose pH was adjusted to 8.5 with a 2 M NaOH solution, after which the solution was stirred for 1 h at 40 °C. The slurry was subsequently centrifuged at 10,000× *g* for 20 min at 4 °C (Sorvall LegendTM XTR., Thermo Scientific, Waltham, MA, USA). The supernatant was treated with dry sodium bisulfite (SBS) to a concentration of 0.01 M, the pH was adjusted to 6.4 with 0.2 M HCl and the mixture was kept in an ice bath overnight. The mixture was then centrifuged at 12,000× *g* for 20 min at 4 °C, after which the supernatant was collected. Solid NaCl was added to the supernatant at a concentration of 0.25 M, and the pH was adjusted to 5.0 with 0.2 M HCl. The mixture was stirred for 30 min at room temperature and then centrifuged at 12,000× *g* for 20 min at 4 °C. The supernatant was subsequently diluted twofold with ice-cold water at pH 4.8 and then centrifuged at 12,000× *g* for 20 min. After centrifugation, the precipitate was washed three times with distilled water, suspended in distilled water (pH 8.0), dialyzed and then lyophilized to obtain 7S globin powder. The 7S protein content was determined to be 94.2% using the bicinchoninic acid (BCA) method. The denaturation temperature value of 7S protein was determined to be 64.53 °C using a differential scanning calorimeter (DSC; Q10, TA Instruments, New Castle, DE, USA), which is close to that of previous reports [10,11], indicating that the degree of denaturation of the 7S protein is relatively low during the extraction process.

### 2.3. Fluorescence Spectroscopy

The interactions between 7S and GA, CA and EGCG were explored by measuring the quenching of protein fluorescence with an F-7000 fluorescence spectrophotometer (Hitachi, Ibaracki, Japan). The experimental method was based on a procedure reported by Zhang et al. [12], with slight modifications. The following instrument settings were used: excitation wavelength of 280 nm, emission wavelength of 290–420 nm, scan rate of 1200 nm/min and excitation and emission bandwidths of 5 nm. The three different polyphenols (GA, CA and EGCG) and 7S globulin were dissolved in phosphate buffer (10 mM, pH 7.0) to prepare polyphenol solutions at different concentrations and a single-concentration 7S protein solution, respectively. The polyphenol solutions at different concentrations were added to the 7S solution and mixed thoroughly to achieve a final 7S concentration of 10 μM and polyphenol concentrations in the range of 0–12 μM. The polyphenol–7S mixtures were measured in a quartz cuvette with a path length of 10 mm at 288, 298 and 308 K, respectively.

Synchronous fluorescence spectrometry was performed under wavelength scanning ranges of 260–340 nm (∆λ = 15 nm) and 220–360 nm (∆λ = 60 nm) at 288 K.

Three-dimensional (3D) fluorescence spectra were measured by setting the excitation and emission wavelengths to 200–350 nm and 220–500 nm, respectively, with a bandwidth of 5 nm and a sampling interval of 10 nm.

### 2.4. CD Analysis

The changes in the secondary and tertiary structure of 7S were analyzed via CD [13]. 7S was dissolved in phosphate buffer (10 mM, pH 7.4) to obtain a 0.3 mg/mL solution, which was injected into a 1 mm quartz sample cell. The sample was analyzed using a MOS-450/AF-CD spectrometer (Biologic, Seyssinet-Pariset, France). The scanning wavelength was set to 190–250 nm for far-UV CD and to 250–320 nm for near-UV CD, and the scanning speed was 1 nm/s. The collected spectral data were analyzed using the online CONTIN program in DichroWeb (http://dichroweb.cryst.bbk.ac.uk/, accessed on 11 September 2023).

### 2.5. Surface Hydrophobicity

Changes in protein surface hydrophobicity were determined with an ANS hydrophobic fluorescent probe. The three different polyphenols (GA, CA and EGCG) and 7S were dissolved in phosphate buffer (10 mM, pH 7.4) to prepare polyphenol solutions at different concentrations and a single-concentration 7S protein solution. The polyphenol solutions at different concentrations were added to the 7S protein solution and mixed thoroughly to achieve a final 7S concentration of 10 μM, with the final concentrations of polyphenols being 0, 0.5, 1, 2, 4, 6, 8 and 10 μM. ANS was dissolved in phosphate buffer to prepare a solution with a concentration of 8 mM for future use. Two milliliters of the combined 7S protein–polyphenol sample solution and 20 μL of ANS solution were mixed, thoroughly homogenized by shaking and allowed to sit for 3 min. The fluorescence intensity of the sample was then determined via an F-7000 instrument with the following settings: excitation wavelength (λex) of 390 nm (Δλ = 5 nm), emission wavelength (λem) of 470 nm (Δλ = 5 nm) and voltage of 500 V. The slope of the linear regression curve between the fluorescence intensity and polyphenol concentration was calculated as the surface hydrophobicity index S_0_.

### 2.6. ITC Measurements

The thermodynamic parameters of the binding of GA, CA and EGCG to 7S were measured with a Malvern MicroCal iTC200 (Malvern, London, UK) microcalorimeter. The experimental method was based on the procedure reported by Budryn et al. [14], with slight modifications. A 10 mM phosphate buffer (pH 7.0) was used to prepare the 7S protein solutions and the GA, CA and EGCG solutions. The protein solution was injected into the sample cell, and the reference cell was injected with distilled water. The polyphenol solution was drawn up with a syringe, the number of titrations was set to 19 and the reaction temperature was set to 25 °C. The corresponding experimental concentrations of the 7S protein and the three polyphenols were 10 μM 7S−5 mM GA, 10 μM 7S−5 mM CA and 5 μM 7S−5 mM EGCG. All of the data were fitted using a one-site model within the ITC v.1 analysis software.

### 2.7. Molecular Docking

The α’ subunit of the soybean 7S protein was selected as a model for molecular docking simulations to observe the interactions of the three polyphenols with the soybean protein visually. The crystal structure of the receptor protein was downloaded from the Research Collaboratory for Structural Bioinformatics (RCSB) database (PDB ID: 1uik), the water hydrating the molecule was removed from the structure and hydrogen atoms were added to prepare the simulation of the receptor protein. For ligand preparation, the two-dimensional (2D) structures of the three ligand molecules were drawn using ChemDraw 14.0 software and saved in cdx format. The ligand structure was subsequently subjected to energy minimization using the MM2 force field in Chem3D 14.0 software and saved in pdb format. Lastly, AutoDockTools was used to assign atom types and calculate partial charges, and the result was saved in pdbqt format for docking. All rotatable bonds were set to be flexible.

### 2.8. Statistical Analysis

The experiments were performed in triplicate, and the data are expressed as the means ± standard deviations (SDs). One-way analysis of variance (ANOVA) and significant difference tests were performed using SPSS 22.0 software (Chicago, IL, USA). Significant differences were determined through one-way analysis of variance (ANOVA) and Duncan’s multiple range test at *p* = 0.05. Differences between means were considered significant when *p* < 0.05.

## 3. Results and Discussion

### 3.1. Fluorescence Quenching Assays

The fluorescence technique is a useful tool for analyzing interactions between small molecules and proteins. The aromatic amino acids such as tryptophan (Trp), tyrosine (Tyr) and phenylalanine (Phe) in 7S have fluorescent properties, which are very sensitive to the polarity of the surrounding microenvironment. Slight alterations in the protein microenvironment caused by protein conformational transitions, biomolecular binding and denaturation can lead to changes in protein intrinsic fluorescence [12].

**Figure 2:**
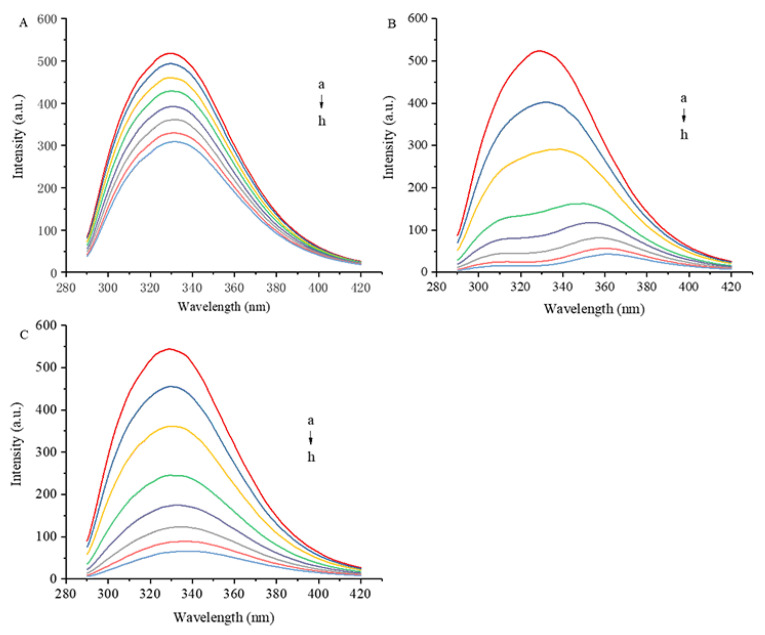
Effect of different concentrations of GA (**A**), CA (**B**) and EGCG (**C**) on fluorescence spectrum of 7S globulin. a–h denotes polyphenol concentrations of 0, 1, 2, 4, 6, 8, 10 and 12 μM, respectively.

As shown in Figure 2, the maximum fluorescence emission wavelength of 7S is 329 nm. The intrinsic fluorescence intensity of 7S decreased as the concentration of polyphenol gradually increased. The maximum concentrations (12 μM) of GA, CA and EGCG reduced the fluorescence intensity of 7S by 40.5%, 89.0% and 87.9%, respectively. The ability of CA and EGCG to quench 7S fluorescence was significantly greater than that of GA, which may be related to the number of hydroxyl groups and the molecular structures of these polyphenols. Xiao et al. [15] concluded that the hydroxylation degree of polyphenols, the position of hydroxyl groups on aromatic rings, the isomers and the esterification of the GA of catechins affect the affinity of polyphenols to proteins. Redshifts and blueshifts in fluorescence spectra indicate decreases and increases, respectively, in the hydrophobicity of the environment surrounding the amino acid residues that intrinsically fluoresce [16]. All three polyphenols caused a redshift in the fluorescence emission peak of 7S, with CA exerting the most significant effect.

### 3.2. Synchronous Fluorescence Spectrometry

**Figure 3:**
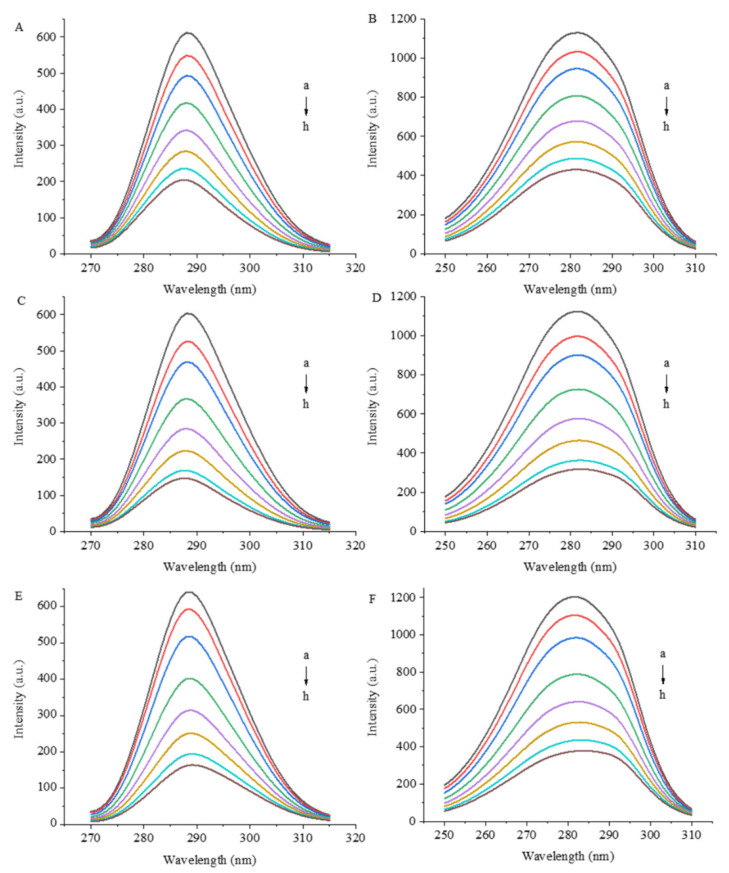
Synchronized fluorescence spectra of 7S globulin interacting with varying concentrations of GA (**A**,**B**), CA (**C**,**D**) and EGCG (**E**,**F**). A/C/E: Δλ = 15 nm, B/D/F: Δλ = 60 nm, a–h denotes polyphenol concentrations of 0, 1, 2, 4, 6, 8, 10 and 12 μM, respectively.

Figure 3 shows the synchronous fluorescence spectra of 7S in the presence of GA, CA and EGCG. Synchronous fluorescence involves the scanning of the excitation and emission wavelengths simultaneously while maintaining a constant wavelength interval (Δλ) between the two wavelengths. When a suitable Δλ is set, synchronous fluorescence spectroscopy reduces spectral overlap by narrowing the spectral band and simplifies complex fluorescence spectra [17]. The fluorescence intensity of a protein is derived mainly from Trp and Tyr residues; when Δλ values are 15 and 60 nm, synchronous fluorescence spectra show fluorescence information for Tyr and Trp residues, respectively.

As the concentration of polyphenols increased, the fluorescence quenching intensity of Trp and Tyr residues increased, indicating that the combination of polyphenols affected the hydrophobicity or structure of the microenvironment of these residues and even had a certain effect on the protein conformation. During the interaction process, the electron density current generated by the redistribution of electron cloud density of polyphenols causes a change in the polarity of the surrounding environment of amino acid residues [18,19] and further affects the fluorescence properties of Trp and Tyr residues. When the polyphenols were present at the greatest concentration (12 μM) in this experiment, the fluorescence intensity of the Trp residues decreased by 66.6%, 75.6% and 74.3% under the influence of GA, CA and EGCG, respectively, and the fluorescence intensity of the Tyr residues decreased by 61.9%, 71.7% and 68.6%, respectively. The fluorescence of the Trp residues of the proteins is more effectively quenched by the three polyphenols than that of the Tyr residues, which may be due to the greater exposure of Trp residues to the surface of the proteins. Koshiyama [20] reported that one molecule of the 7S protein contains three Trp residues, each of which is located on the surface of the protein, and their photophysical properties are extremely sensitive to the surrounding environment. The fluorescence intensity of Trp residues is particularly affected by alterations in protein conformation, changes in the polarity of the fluorophore microenvironment and the effects of hydrogen bonds and other noncovalent interactions [21,22].

### 3.3. 3D Fluorescence Spectra

3D fluorescence spectroscopy was used to scan the 7S–polyphenol complexes, and the effects of GA, CA and EGCG on the amino acid residues and the peptide chain conformation of 7S were further investigated by constructing a 3D fluorescence contour plot.

**Figure 4:**
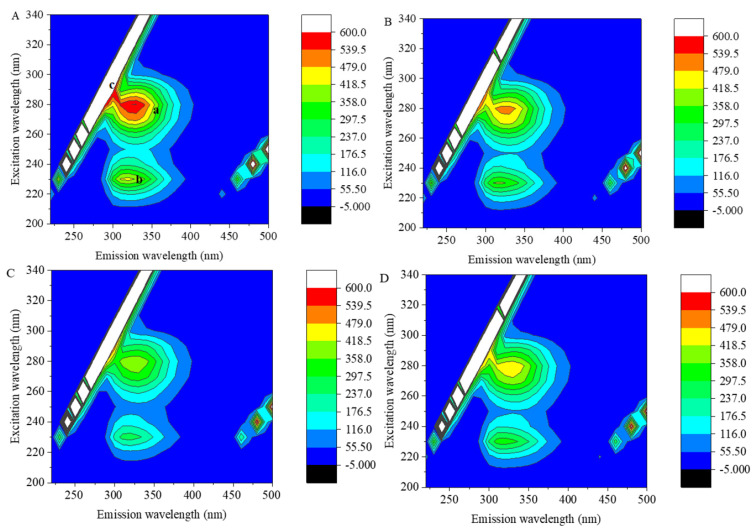
Three − dimensional fluorescence contour plots of 7S globulin (**A**) and the 7S−GA (**B**), 7S−CA (**C**) and 7S−EGCG (**D**) systems. Peak a and peak b are the characteristic peaks of protein 3D fluorescence spectra, peak c is the Ray-leigh scattering peak.

Figure 4 shows the 3D fluorescence contour plots of 7S in the presence or absence of GA, CA and EGCG at concentrations of 1:1. Peak a and peak b are the characteristic peaks of protein 3D fluorescence spectra; these peaks represent the relevant spectral information for the amino acid residues with fluorescence properties and the structures of polypeptide chains, respectively [23], and their intensity is correlated with the secondary structure of the protein. Peak c is the Rayleigh scattering peak.

As shown in Figure 4A, the fluorescence intensities of 7S peak a (λex/λem: 280 nm/330 nm) and peak b (λex/λem: 230 nm/320 nm) were 602.8 and 442.5, respectively. After the addition of polyphenols, owing to the effects of polyphenols on the Trp and Tyr residues of 7S globulin and their microenvironment, the fluorescence intensities of peaks a and b decreased, the color of the characteristic peaks in the graph became lighter and the contour lines became sparse. After the addition of GA, CA and EGCG, the fluorescence intensities of peak a decreased to 510.3, 412.4 and 476.1, respectively, and the intensities of peak b decreased to 371.4, 270.4 and 335.7, respectively. Structural changes in a protein can shift the fluorescent amino acid residues originally located on the surface to the interior of the protein [24], and the binding of polyphenols can cover fluorescent amino acid residues, both of which can reduce the fluorescence intensity of peak a. When polyphenols interacted with the 7S protein, it may have caused a change in the conformation of the peptide chain, resulting in a decrease in the fluorescence intensity of peak b.

### 3.4. Mechanism of Fluorescence Quenching

The type and mechanism of fluorescence quenching are usually determined according to the Stern–Volmer equation, which is calculated as follows [25]:

F_0_/F = 1 + *K_Q_*τ_0_[*Q*] = 1 + *K_SV_*[*Q*] (1)

In this equation, F_0_ and F are the fluorescence intensities measured in the presence or absence of the quencher, respectively; *K_Q_* is the biomolecular quenching rate constant and τ_0_ is the average fluorescence lifetime of the biomolecule without the quencher, the value of which is usually 10^−8^ s.

For the static quenching, the binding constant of the interaction between polyphenols and 7S and the number of binding sites per protein could be calculated according to a double-logarithmic equation [26]:

log[(F_0_ − F)/F] = log*Ka* + *n*log[*Q*] (2)

**Figure 5:**
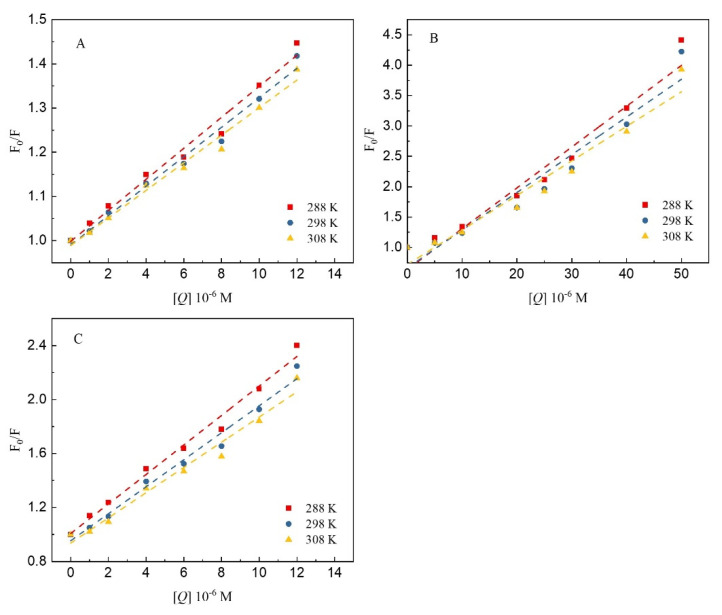
The Stern–Volmer plots for the quenching of 7S globulin by GA (**A**), CA (**B**) and EGCG (**C**) at different temperatures (288, 298 and 308 K).

Figure 5 shows Stern–Volmer plots of the fluorescence quenching of 7S by the three polyphenols at different temperatures. Furthermore, Table 1 shows the quenching constants of the three polyphenols when bound to 7S. As the temperature increased, the value of *K_SV_* for GA, CA and EGCG binding to 7S increased, and the value of *K_Q_* was greater than the maximum diffusion collision quenching constant value—2.0 × 10^12^ L/mol/s. The linearity of the Stern–Volmer regression curve indicates that the effects of these three polyphenols on 7S were mediated by static quenching, with the effect of temperature on the interaction between the polyphenols and protein being greater. A consistent conclusion was reported by Jia et al. [26] in a study of the interactions of different polyphenols with β-lactoglobulin. At 308 K, the quenching constants of CA were higher than those of GA and EGCG, indicating that the quenching effect of CA on 7S fluorescence was stronger than that of the other two polyphenols. The numbers of hydroxyl groups and the molecular structures of polyphenols play important roles in their binding to proteins [15]. Compared with GA and CA, EGCG has a larger 3D molecular structure, preventing it from easily reaching equilibration among the binding sites on 7S and resulting in a quenching constant that is lower than that of CA, which is consistent with the findings reported by Al-Hanish et al. [27]. In addition, phenolic acids exist in solution because of deprotonation under neutral conditions, and the binding affinity of phenolic acids can increase via electrostatic interactions with 7S, which may also explain why the quenching constant value of CA is greater than that of the other tested polyphenols.

### 3.5. Changes in the Secondary and Tertiary Structure of 7S

Since the results of fluorescence experiments show that the binding of polyphenols may affect the conformation of proteins, the changes in the secondary and tertiary structures of proteins are determined using CD spectroscopy. Changes in the contents of α-helixes, β-sheets, β-turns and random coils in the secondary structure of 7S were determined in the far-UV wavelength range, the data of which are presented in Table 2.

After 7S binds with any one of the three tested polyphenols via noncovalent bonds, the structure of the 7S protein has fewer α-helixes but a greater number of random coils compared with the structure of the 7S protein alone. Thus, the binding of the three polyphenols exerts a substantial effect on the secondary structure of the protein by converting α-helixes into random coils, with the structure of 7S becoming loosely unfolded. Hasni et al. [28] reached a similar conclusion when studying the interaction between casein and tea polyphenols. After a polyphenol binds to the protein, its hydroxyl group forms hydrogen bonds with the N-H, C=O and C-N residues of the protein, allowing a rearrangement of the inner hydrogen bond network, thus leading to the changes observed in the secondary structure of 7S. Liu et al. [29] reported that after zein combined with different polyphenols, the secondary structure of the protein was altered according to the influence of the polyphenol type and binding mode. The research of Li et al. [30] indicated that the hydrogen bonds and hydrophobic interaction of tea polyphenols may cause changes in the secondary structure of the protein.

**Figure 6:**
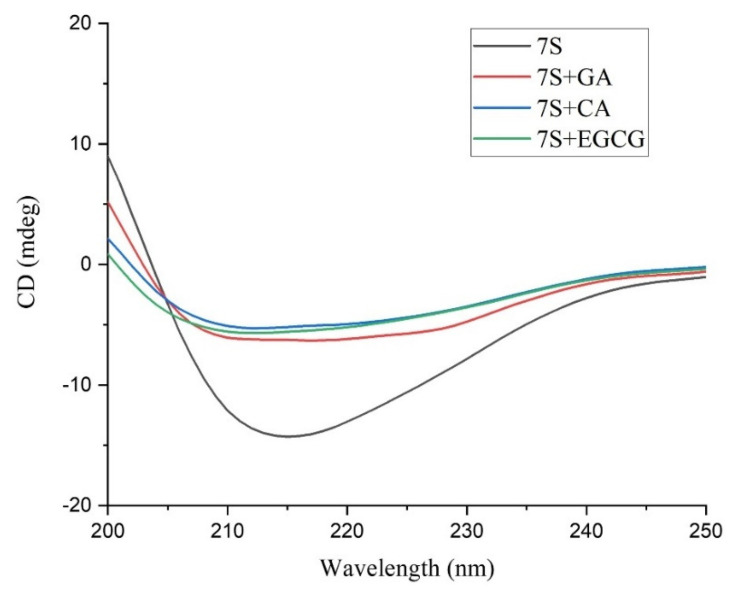
The far −UV CD spectra of 7S globulin and GA/CA/EGCG complexes.

Information on the 7S tertiary structure after the addition of polyphenols was obtained by analyzing the data measured in the near-UV wavelength range. In this wavelength range, the main chromophores are aromatic amino acids (Trp, Tyr, Phe) and disulfide bonds [31]. As shown in Figure 6, compared with that of 7S, the molar ellipticity of the 7S−EGCG complex is lower, and the positive peak in the range of 256–293 nm is reduced. Most of the molar ellipticities in its CD spectroscopy data are negative, indicating that the microenvironment of aromatic amino acids has changed. However, there was minimal change in the molar ellipticity of 7S when combined with GA and CA.

### 3.6. Surface Hydrophobicity

The effects of the interactions of the different polyphenols with 7S on the surface hydrophobicity (S_0_) of the protein were determined, and the data are shown in Table 2. ANS binds to nonpolar regions of proteins through noncovalent interactions, and a greater fluorescence intensity of the complex indicates a stronger surface hydrophobicity of the protein.

The surface hydrophobicity of 7S was reduced after the addition of GA, CA and EGCG. It is very clear that the surface hydrophobicity of 7S decreased in the presence of the three polyphenols because the binding of the polyphenols increased the number of polar groups, such as hydroxyl groups and carboxyl groups. This increase changed the polarity of the environment surrounding 7S, and the hydrophilicity of the protein increased. For GA, CA and EGCG, the surface hydrophobicity of 7S decreased as the complexity of the polyphenol structure and the number of hydroxyl groups increased, indicating that the polyphenol structure has a strong effect on the surface hydrophobicity of the protein. In addition, polyphenols tend to interact with free hydrophobic amino acids on protein surfaces, which reduces the number of hydrophobic residues and subsequently reduces the hydrophobicity of the protein; the binding of polyphenols can also occupy ANS-binding sites on proteins [32], which in turn reduces the fluorescence intensity and surface hydrophobicity of the ANS-protein complex. Moreover, the change in the structure of the protein causes changes in the hydrophilic and hydrophobic regions inside and outside the globulin structure, which also explains the decrease in the surface hydrophobicity of the protein.

### 3.7. ITC

ITC is a powerful analytical method for measuring the affinity and thermodynamic parameters of interactions between any two molecules. Noncovalent interactions occurring at molecular interfaces will exhibit specific thermodynamic parameters, and the hypothesized driving force of intermolecular interactions is mainly based on the positive and negative values of these parameters. A negative Gibbs free energy (Δ*G* < 0) indicates that the intermolecular interactions are spontaneous. In this case, the change in Gibbs free energy (Δ*G* = Δ*H* − *T*Δ*S*) is influenced by both enthalpy and entropy. The positive or negative values of enthalpy and entropy indicate their contributions to the negative changes in Gibbs free energy. Negative binding enthalpy and positive binding entropy favor intermolecular interactions. Changes in enthalpy and entropy can reveal the forces and mechanisms that drive the formation of intermolecular interactions. Thermodynamic information can be used to characterize conformational changes, hydrogen bonding, hydrophobic interactions and interactions between charged residues in proteins.

**Figure 7:**
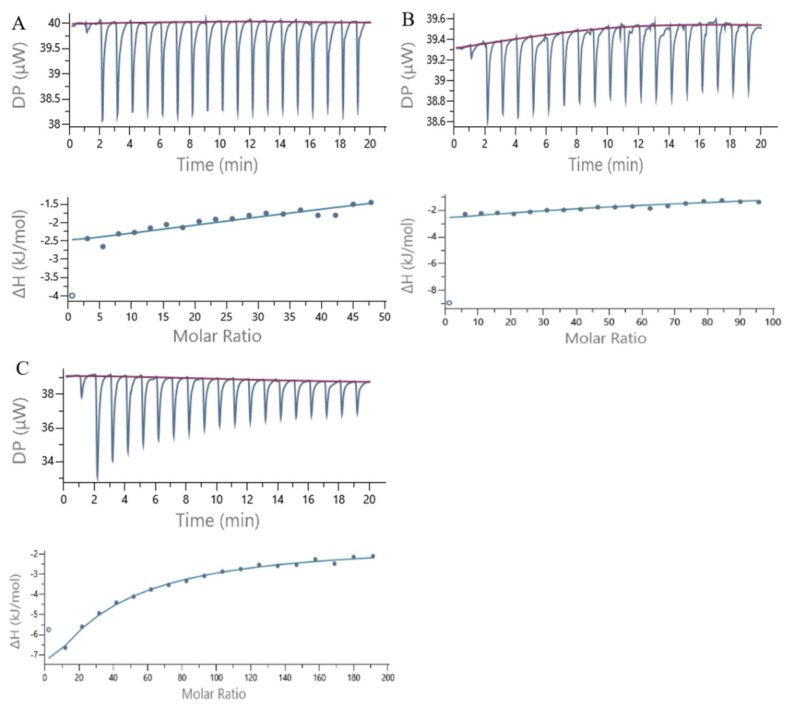
ITC data from the titration of 7S globulin with GA (**A**), CA (**B**) and EGCG (**C**) at 25 °C. The amount of heat measured per injection vs. time and also the amount of heat measured per mole of the injected polyphenol against the molar ratio of polyphenol to 7S for each injection are shown at the top and bottom.

Figure 7 shows a plot of the ITC heat flow profile and a single point fitting plot for the noncovalent interactions of 7S with GA, CA and EGCG. As shown in Figure 7, significant exothermic reactions occurred when GA, CA and EGCG were added to the 7S solution in a dropwise manner, reflected by the exothermic peaks in the figure. As the polyphenols were continuously added in a dropwise manner, the amount of heat released from the reaction gradually decreased until it remained stable. Table 3 shows the thermodynamic parameters obtained from ITC. *N* represents the stoichiometric number, and *K_D_* represents the binding constant. Δ*H* is the change in enthalpy, which mainly reflects the changes in hydrogen bonds and ionic interactions, and Δ*S* is the change in entropy, which reflects the changes in hydrophobic interactions and changes in the protein conformational freedom.

The correlations of enthalpy changes with molar ratios for polyphenols/protein binding interactions were fitted using the single-site binding model, from which the dissociation of the polyphenol–protein complex (*K_D_*) was obtained. A smaller magnitude of *K_D_* indicates a stronger intermolecular binding affinity. On the basis of the data presented in Table 3, among the three polyphenols, CA has the strongest binding affinity for 7S. All the *K_D_* values of the three polyphenols for binding to 7S are on the order of 10^−4^, indicating weak binding [33]. Thus, theoretically, greater concentrations of ligands are needed to saturate the binding sites of the receptor protein; this phenomenon may be due to the substantial differences in the molecular weights between 7S and the three polyphenols.

In terms of the changes in entropy and enthalpy, Δ*H* < 0 and −*T*Δ*S* > 0 for EGCG and CA, indicating that hydrogen bonds and van der Waals forces are the main driving forces promoting the binding of EGCG and CA to 7S [34]. Moreover, both of the binding processes are driven by favorable enthalpy and unfavorable entropy. Therefore, the change in enthalpy has a greater contribution to Δ*G*.

For GA, the main source of the Δ*G* value is derived from Δ*S*, implying the importance of hydrophobic interaction. Δ*H* < 0 and Δ*S* > 0 are characteristics of electrostatic interaction, which indicates that the electrostatic interaction is dominant over hydrogen bond and van der Waals interactions for the binding of GA to 7S [35]. This may be related to the smaller molecular weight of GA. The interaction between polyphenols with larger molecular weight and 7S is greater, which is consistent with the conclusion of Frazier et al.’s report [36]. Δ*G* < 0 for the interactions of the three polyphenols with the 7S protein, indicating that the three polyphenols spontaneously bind to 7S.

In Table 3, the values of *N* indicate that 1 mol of 7S binds with 10.0 mol of GA, 7.23 mol of CA and 1.68 mol of EGCG, and all three polyphenols are bound to 7S at multiple sites. As the molecular weights and complexity of the molecular structures of GA, CA and EGCG increase, *N* decreases accordingly. Thus, the amount of bound polyphenols is substantially affected by the physicochemical properties of the polyphenols themselves. The binding amount of EGCG to 7S is the lowest among the three polyphenols, which is related to its larger molecular weight, more complex steric structure, and the presence of multiple −OH and aromatic rings that potentially serve as binding sites. In addition, the amount of bound polyphenols is also affected by the receptor protein. Multiple basic amino acid residues and proline residues on the same protein molecule simultaneously bind polyphenols, and the protein structure also affects the interactions [37].

Comparison of the detailed fluorescence and ITC thermodynamic binding parameters revealed that the K and n values determined through ITC were higher than those determined in the fluorescence experiments. This may be due to the fact that fluorescence quenching characterized protein–polyphenol interactions occurring in the environment surrounding Trp, whereas ITC determined all protein–polyphenol intermolecular interactions occurring at different sites of the protein [38].

### 3.8. Molecular Docking

The interactions between the soybean protein and the three polyphenols were calculated and simulated using software. The results of the simulation visually show the binding of polyphenols to the soybean protein and the types of forces involved. Some amino acid sequence similarity has been observed between the subunits of 7S [39]; therefore, the α’ subunit of 7S was selected as the receptor protein model.

**Figure 8:**
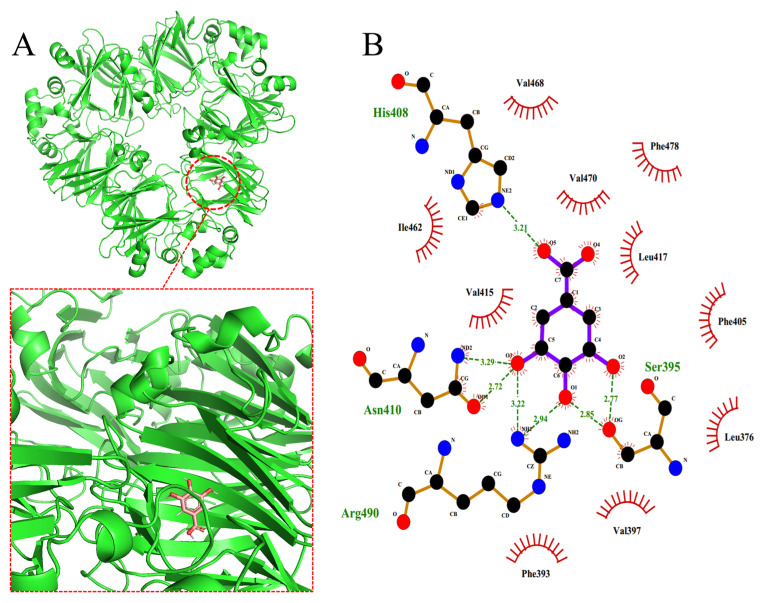

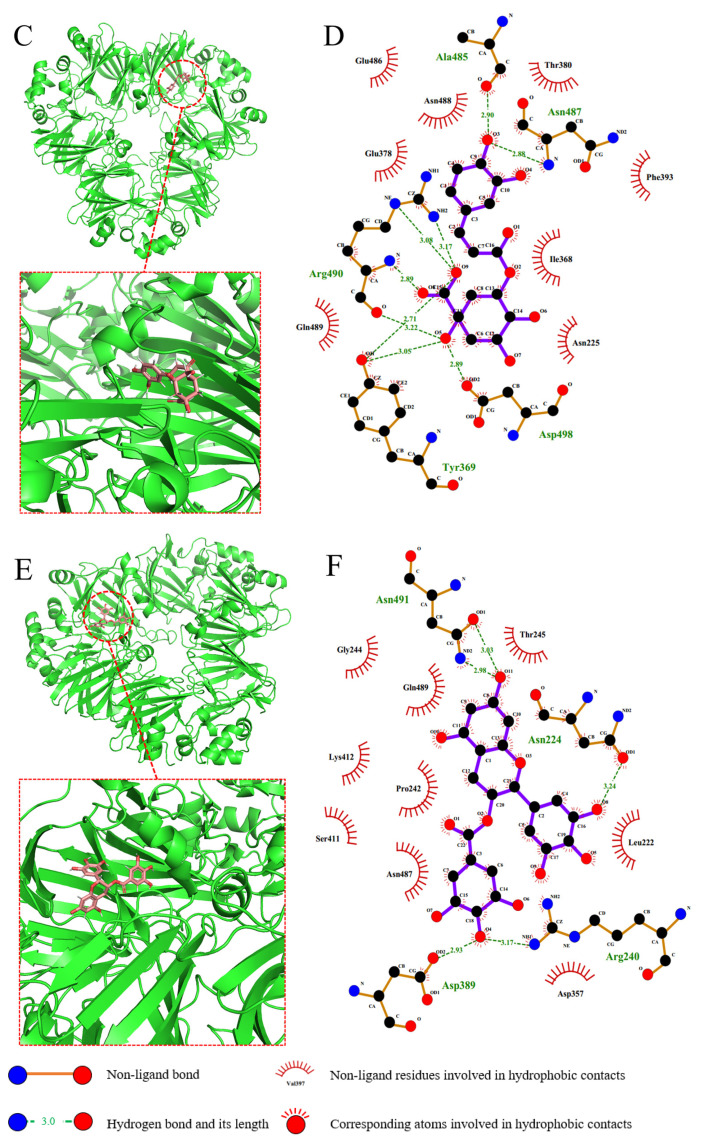
Molecular docking of GA (**A**), CA (**C**) and EGCG (**E**) into α’subunits of 7S globulin. Proteins and polyphenols are shown in green and red. (**B**,**D**,**F**) are two−dimensional interaction analysis of GA, CA, and EGCG with 7S protein, respectively.

Figure 8 shows the optimal molecular docking simulation results for GA, CA and EGCG bound to the α’ subunit of 7S. Table 4 summarizes the donor atoms and the acceptor atoms of the hydrogen bonds that formed upon the binding of the three polyphenols to the α’ subunit of 7S in Figure 8. The formation of hydrogen bonds is attributed mainly to the interaction between the hydroxyl groups of polyphenols and the oxygen atoms of the carboxyl moiety or the nitrogen atoms of the amino group, imino group and heterocyclic ring of the amino acid residues.

On the basis of the software simulation analysis, the binding energy of GA to the receptor protein is −6.6 kcal/mol. As shown in Figure 8A,B, GA binds to the protein in a hydrophobic pocket and interacts mainly with the following hydrophobic residues: Leu-376, Phe-393, Val-397, Phe-405, Val-415, Leu-417, Ile-462, Val-468, Val-470 and Phe-478. GA interacts with these residues through stronger hydrophobic interactions and van der Waals forces. In addition, the hydroxyl group of GA also forms hydrogen bonds with the surrounding amino acids. The binding energy of CA to the receptor protein is −7.5 kcal/mol, and its binding site is located within a pocket formed by both hydrophilic and hydrophobic residues. As shown in Figure 8C,D, the amino acid residues that interact with CA are mainly Phe-393, Ile-368, Tyr-369, Glu-486, Asn-488, Glu-378, Asn-487 and Asp-498. CA interacts with the receptor protein through hydrophobic interactions, van der Waals forces and electrostatic interactions. The hydroxyl and carboxyl groups of CA form multiple hydrogen bonds with the amino acids within the binding pocket, playing a key role in stabilizing its binding. The binding energy of EGCG to the receptor protein is −9.5 kcal/mol. As shown in Figure 8E,F, fewer hydrophobic residues are located in the region to which EGCG binds, and most are hydrophilic residues. The hydrogen bonds between the hydroxyl group on the aromatic ring of EGCG and the surrounding amino acid residues are key factors that contribute to sustaining the binding of EGCG to the receptor protein. In addition, van der Waals forces and electrostatic interactions also contribute to the interaction between EGCG and the protein.

When the results of the molecular docking simulation experiments were compared with those of the ITC thermodynamic data, the differences in the results were related to those of the software simulation algorithm and the rigidity and flexibility of the polyphenol molecules. The differences between the theoretical structures and the actual spatial structures of the protein and the polyphenols in solution can also affect the data. In Figure 8, the binding sites of the three polyphenols in the α’ subunit of 7S are not identical, and GA and CA tend to bind to the hydrophobic regions inside 7S. Hasni et al. [28] reported that the binding sites of different polyphenols within the same protein are not the same, which is consistent with the findings of the present experiment. In previous studies [40], hydrophobic amino acid residues of receptor proteins were always involved in the binding of EGCG, which contradicts the docking simulation results in the present study. This difference may be related to the different kinds of proteins used in the studies. The α’ subunit of 7S has a complex and compact structure and contains fewer hydrophobic amino acids than other subunits do, while the EGCG molecule has a larger spatial structure and is unable to access and bind to easily the hydrophobic pocket inside the receptor protein. However, EGCG has a strong affinity, which is consistent with the results of the fluorescence spectroscopy experiment. The decrease in the intensity of the fluorescence spectrum of EGCG indicates that this compound interacts with hydrophobic amino acid residues with fluorescence characteristics in 7S. Therefore, we speculated that a hydrophobic interaction occurs between EGCG and 7S.

## 4. Conclusions

In this study, the noncovalent interactions between three polyphenols (GA, CA and EGCG) and 7S were investigated via multispectral, thermodynamic and molecular docking techniques. The results showed that hydrogen bonds and van der Waals forces are the main driving forces promoting the binding of EGCG and CA to 7S. Electrostatic interaction plays an important role in the combination of GA. The binding forces of the three polyphenols are different, which may be affected by the molecular weights of the polyphenols. The binding of the polyphenols affects both the microenvironment of Trp and Tyr residues and the conformation of the polypeptide chain of 7S, which leads to the change in 7S fluorescence characteristics. The binding of GA and EGCG induces the static quenching of 7S fluorescence. CA has the greatest binding affinity to 7S, and multiple mechanisms are involved in its quenching effect on 7S fluorescence. The noncovalent interaction results in the conversion of β-sheets to random coils, the protein structure is loosely unfolded and the surface hydrophobicity of 7S is reduced. The results of molecular docking showed that polyphenols with larger molecular weights and more hydroxyl groups have greater binding affinity for 7S. However, the large spatial structure also limits the binding sites on the protein. The interaction mechanism and difference between polyphenols and 7S were comprehensively discussed in this work. The research results can provide theoretical support for the use of noncovalent complexes of soybean protein and polyphenols as functional ingredients in food.

## Figures and Tables

**Table 1:** The quenching and binding constants for the interaction of 7S globulin with GA/CA/EGCG at different temperatures.

Compound	T (K)	*K_SV_* (10^4^ L/mol)	*n*	*K_a_* (10^4^ L/mol)
7S−GA	308	0.40 ± 0.01 ^a^	0.94 ± 0.04 ^a^	4.21 ± 0.22 ^a^
	298	0.42 ± 0.02 ^ab^	1.12 ± 0.06 ^b^	5.10 ± 0.30 ^b^
	288	0.44 ± 0.02 ^b^	1.18 ± 0.06 ^b^	5.37 ± 0.30 ^b^
7S−CA	308	5.69 ± 0.03 ^a^	1.17 ± 0.06 ^a^	6.23 ± 0.32 ^a^
	298	6.21 ± 0.17 ^b^	1.42 ± 0.05 ^b^	7.44 ± 0.32 ^b^
	288	6.72 ± 0.13 ^c^	1.36 ± 0.06 ^b^	7.09 ± 0.31 ^b^
7S−EGCG	308	2.35 ± 0.03 ^a^	0.90 ± 0.04 ^a^	4.54 ± 0.20 ^a^
	298	2.53 ± 0.01 ^b^	1.25 ± 0.06 ^b^	6.23 ± 0.30 ^b^
	288	2.77 ± 0.01 ^c^	1.52 ± 0.11 ^c^	7.57 ± 0.56 ^c^

Different letters in the same column of the same protein indicate significant differences (*p* < 0.05).

**Table 2:** Secondary structure and surface hydrophobicity of 7S with GA, CA and EGCG.

Sample	Secondary Structure Content (%)				Surface Hydrophobicity
	α-Helixes	β-Sheets	β-Turns	Random Coils	S_0_
7S	22.6 ± 0.2 ^c^	36.2 ± 0.6 ^b^	16.3 ± 0.5 ^c^	24.9 ± 0.2 ^a^	56.80 ± 1.23 ^d^
7S−GA	20.7 ± 0.1 ^b^	37.2 ± 0.4 ^b^	12.7 ± 0.0 ^a^	29.4 ± 0.6 ^b^	45.57 ± 0.65 ^c^
7S−CA	17.9 ± 0.3 ^a^	33.3 ± 0.2 ^a^	18.3 ± 0.4 ^d^	30.5 ± 0.1 ^b^	41.59 ± 0.81 ^b^
7S−EGCG	18.5 ± 0.2 ^ab^	34.7 ± 0.1 ^a^	14.0 ± 0.2 ^b^	32.8 ± 0.1 ^c^	25.97 ± 0.53 ^a^

Values are expressed as the mean ± SD. Different letters in the same column of the same protein indicate significant differences (*p* < 0.05). The values of surface hydrophobicity are the averages of three measurements (*n* = 3).

**Table 3:** Thermodynamic binding parameters for the interactions of GA/CA/EGCG with 7S globulin.

Compounds	*N* (Sites)	*K_D_* (M)	Δ*H* (kJ/mol)	Δ*G* (kJ/mol)	*T*Δ*S* (kJ/mol)
7S−GA	10.0 ± 0.40 ^c^	7.20 × 10^−4^ ± 2.91 × 10^−5 b^	−3.66 ± 0.22 ^c^	−18.0	−14.3
7S−CA	7.23 ± 0.75 ^b^	4.44 × 10^−4^ ± 1.75 × 10^−5 a^	−162 ± 4.62 ^b^	−13.4	148
7S−EGCG	1.68 ± 0.38 ^a^	4.85 × 10^−4^ ± 1.35 × 10^−5 a^	−335 ± 17.67 ^a^	−18.9	316

Different letters in the same column of the same protein indicate significant differences (*p* < 0.05).

**Table 4:** Atoms and hydrogen bond lengths between GA/CA/EGCG and α’subunits of 7S globulin.

Polyphenol	Donor Atom	Acceptor Atom	Hydrogen Bond Length (Å)
GA	NE2 (His-408)	O5 (GA)	3.21
	O3 (GA)	OD1 (Asn-410)	2.72
	ND2 (Asn-410)	O3 (GA)	3.29
	NH1 (Arg-490)	O3 (GA)	2.94
	NH1 (Arg-490)	O1 (GA)	2.94
	OG (Ser-395)	O1 (GA)	2.85
	O2 (GA)	OG (Ser-395)	2.77
CA	O3 (CA)	O (Ala-485)	2.90
	N (Asn-487)	O3 (CA)	2.88
	NH2 (Arg-490)	O9 (CA)	3.17
	OH (Tyr-369)	O9 (CA)	2.71
	N (Arg-490)	O8 (CA)	2.89
	O5 (CA)	O (Arg-490)	3.22
	O5 (CA)	OD2 (Asp-498)	2.89
EGCG	O11 (EGCG)	OD1 (Asn-491)	3.03
	ND2 (Asn-491)	O11 (EGCG)	2.98
	O8 (EGCG)	OD1 (Asn-224)	3.24
	O4 (EGCG)	OD2 (Asp-389)	2.93
	NH1 (Arg-240)	O4 (EGCG)	3.17

## Data Availability

All the data presented in this study are available within the article.
